# Clinical significance and prognostic value of TRIM24 expression in esophageal squamous cell carcinoma

**DOI:** 10.18632/aging.101037

**Published:** 2016-09-28

**Authors:** Jun Chi, Qing Yang, Xiao-Feng Xie, Xian-Zi Yang, Mei-Yin Zhang, Hui-Yun Wang, Guo-Liang Xu

**Affiliations:** ^1^ Department of Endoscopy and Laser, Sun Yat-Sen University Cancer Center, Guangzhou 510060, China; ^2^ State Key Laboratory of Oncology in South China, Sun Yat-Sen University Cancer Center, Guangzhou 510060, China; ^3^ Guangdong Esophageal Cancer Institute, Guangzhou 510060, China; ^4^ Collaborative Innovation Center for Cancer Medicine, Guangzhou 510060, China

**Keywords:** TRIM24, RARα, esophageal squamous cell cancer, prognosis

## Abstract

Tripartite motif-containing 24 (TRIM24), a member of the transcription intermediary factor 1 family, is defined as a co-regulator with several nuclear receptors, such as RARα. TRIM24 has been reported to be involved in many cancers. In this study, we aimed to investigate the expression pattern and prognostic significance of TRIM24 and its relationship with RARα in esophageal squamous cell cancer (ESCC). Both mRNA and protein expression levels of TRIM24 were found to be significantly decreased in ESCC, as judged by qRT-PCR and western blot. Immunohistochemistry staining shows that the reduced TRIM24 protein is associated with lymph node metastasis (*P*=0.024), advance pathological TNM (pTNM) stage (*P*=0.046) and recurrence/metastasis (*P*=0.001). Upregulated TRIM24 protein predicts longer overall survival and disease-free survival (both *P*<0.001) and is an independent predictor for good prognosis (HR, 0.519; 95%CI, 0.341-0.788; *P*=0.002). TRIM24 expression has been proven remarkably to improve prediction of survival of pTNM stage in ESCC patients, especially in stage I and II. However, no significant relationship was found between TRIM24 and RARα expression levels. In conclusion, reduced TRIM24 protein is associated with poor survival in ESCC patients, suggesting TRIM24 protein is a potential prognostic biomarker for ESCC.

## INTRODUCTION

Esophageal cancer (EC) is one of the most common malignancies worldwide, with approximately 455,800 new cases and 400,200 deaths in 2012 [1]. In China, which is known as a high-incidence area, EC is one of the leading causes of cancer-related death [2], and most tumors (up to 90%) are esophageal squamous cell carcinomas (ESCC). Due to delayed diagnosis and early metastasis, the overall 5-year survival rate of patients with ES ranges from 15% to 25% despite of the advances in therapeutic techniques [3]. Even in patients with curative treatment (resection with or without neo-adjuvant therapy), the 5-year survival rate is only 35% – 45% [4]. Currently, TNM staging system is the most important tool for determining appropriate treatment and predicting survival. However, a completely different prognosis and response to the same chemoradiotherapy (CRT) may be present in patients with the same TNM stage due to the large variability and heterogeneity of tumor cells. As we know, tumor biological behavior is controlled by their activated molecular signaling pathways. At present, no molecular biomarker for ESCC is available in clinical routine practice. Therefore, it is of great clinical value to identify new molecular biomarkers for improving diagnosis, prognosis and response to CRT in the patients with esophageal cancer.

Tripartite motif-containing 24 (TRIM24), also called transcription intermediary factor 1-alpha (TIF1α), consists of an amino-terminal RING-B boxes-coiled coil (RBCC/TRIM) motif, a carboxy-terminal Plant Homeodomain (PHD) finger-bromodomain unit and a nuclear receptor interaction box (NR box or LXXLL motif) [5-7]. As a member of the tripartite motif (TRIM) protein family, which is defined as E3 ubiquitin ligases, TRIM24 can ubiquitylate and negatively regulates the level of p53 [8, 9]. TRIM24 is also identified as a co-regulator with several nuclear receptors, such as retinoic acid (RARs), retinoic acid X (RXRs), vitamin D3 (VDR), estrogen and progesterone receptors [10]. It was reported that TRIM24 could activate estrogen-dependent genes associated with cellular proliferation and tumor development in breast cancer, and was negatively correlated with survival of this disease [11, 12]. Recent studies reveal that TRIM24 is upregulated in glioblastoma and gastric cancer, and promotes tumor growth and induces chemotherapy resistance via the phosphatidylinositide 3-kinase (PI3K)/Akt pathway [13, 14]. However, some other studies show that mice with deletion of TRIM24 are predisposed to spontaneous hepatocarcinogenesis with the increased oncogenic activity of RARα, demonstrating that TRIM24 gene functions as a tumor suppressor while RARα as an oncogene in the development and progression of liver cancer [15-17]. Altogether, the role of TRIM24 in carcinogenesis differs in different tissue types. However, its expression pattern in esophageal squamous cell cancer had not yet been defined.

As a co-regulator associated with TRIM24, RARα is a subtype of retinoic acid receptors (RARs), a family of zinc finger transcription factors known as nuclear receptors [18]. RARα was involved in the carcinogenesis of several types of cancers [19-22]. Of these studies, some showed that the overexpressed RARα is an unfavorable prognostic factor in oral squamous cell cancer [20] and ovarian cancer [21], but is an indicator of favorable prognosis in gastric cancer [22]. Studies also indicate that RARα expression level is increased gradually from normal, premalignant to malignant (including both adenocarcinoma and SCC) esophageal tissues [23, 24]. However, the relationship between TRIM24 and RARα and their interaction have not been elucidated in ESCC.

In the current study, we aimed to investigate 1) TRIM24 expression level and its relationship with RARα expression in ESCC; 2) the correlation between TRIM24 expression and clinicopathological characteristics in ESCC patients; 3) the prognostic value of TRIM24 in ESCC patients.

## RESULTS

### TRIM24 mRNA and protein levels were significantly decreased in ESCC tissues

**Figure 1:**
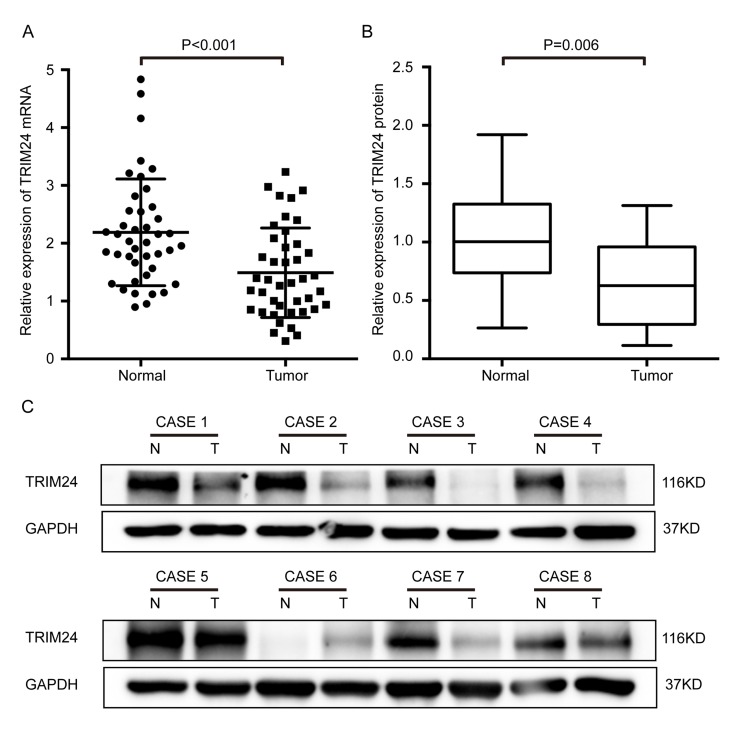
Expression levels of both TRIM24 protein and mRNA were significantly lower in ESCC tissues than in the matched adjacent non-cancerous tissues (NCET) of the same patients (**A**) Comparison of the relative expression levels of TRIM24 mRNA in 42 pairs of ESCC and NCET samples, which were examined by RT-PCR (mean ± SD, Paired t test, *P*< 0.001). (**B**) Comparison of the expression levels of TRIM24 protein in 19 pairs of ESCCs and the matched NCETs, which were detected by Western blot. (**C**) Protein levels of TRIM24 in eight representative ESCC tissues (T) and paired adjacent NCET (N) were analyzed by Western blotting.

To explore the clinical significance of TRIM24, we first determined its mRNA expression in 42 ESCC samples by quantitative RT-PCR. The result shows that TRIM24 mRNA level in the cancer tissues is significantly lower (*P*<0.001, Fig. 1A) than that in the adjacent non-cancerous esophageal tissues (NCET). However, no relationship was found between the mRNA expression and clinical features in ESCC patients (Supplemental Table 1) because the samples size is small. In order to confirm whether TRIM24 protein is also decreased, we employed Western blot to analyze 19 pairs of ESCC tissues and matched NCET (randomly selected from the 42 ESCC). The results demonstrate that the protein level of TRIM24 in ESCC tissues is indeed reduced in about 73.7% (14/19) of these ESCCs (*P*= 0.006, Fig. 1B and 1C), which is concordant with TRIM24 mRNA in ESCC.

**Figure 2:**
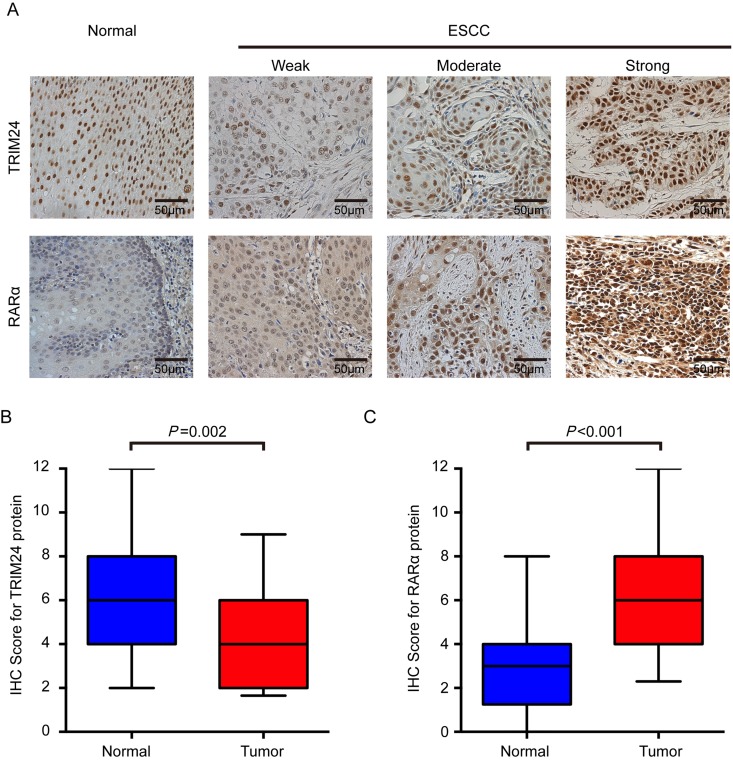
TRIM24 and RARα protein expression in ESCCs and adjacent NCETs (**A**) Representative immunohistochemistry images for TRIM24 (upper panel) and RARα (lower panel) protein expression were presented. Upper panel: The first image represents the high expression of TRIM24 in an adjacent NCET, and then weak, moderate and strong expressions of TRIM24 protein in ESCC tissues. Lower panel: The first image represents the very weak expression of RARα in adjacent non-cancerous tissues, and then weak, moderate and strong expressions of RARα in ESCC tissues. All the images were shot at 200x magnification. (**B**) and (**C**) TRIM24 and RARα protein expression levels were compared between ESCC and adjacent NCET specimens. Statistical analyses were performed by Paired-Samples *t-*test.

To further investigate the clinical significance of TRIM24, we performed immunohistochemical staining of TRIM24 protein in 213 formalin-fixed paraffin-embedded (FFPE) ESCC specimens. As shown in Figure 2B, TRIM24 protein is predominantly localized in the nuclear compartments, with the strongest staining in the normal esophageal epithelium cell, especially in basal and suprabasal layers. Compared with the NCET tissues, the ESCC tissues exhibit significantly lower expression level of TRIM24 protein (*P* = 0.002, Fig. 2A), which is concordant the results of RT-PCR and Western blot analysis on fresh ESCC tissues.

### Relationship between TRIM24 and clinicopathological characteristics in ESCC

TRIM24 protein is reduced in ESCC, which implies that it functions as a tumor suppressor in ESCC. To elucidate the clinical significance of the TRIM24 protein in ESCC, we first divided all patients into a low (n=137) or high (n=76) expression group based on the cutoff value of 4 immunoreactive scores (IRS) for TRIM24 staining. Chi-square test was used to analyze the relationship between TRIM24 expression and clinicopathological characteristics. The results demonstrate that 71.6% (78/109) of patients with low TRIM24 expression have lymph node metastasis (LNM) while only 28.4% (31/109) of patients with high expression have LNM (*P*=0.024, Table 1); 71.4% (70/98) of ones with low expression have pathological tumour-node-metastasis stage (pTNM stage) III while only 28.6% (28/98) of ones with high expression have pTNM stage III (*P*=0.046, Table 1), suggesting that TRIM24 may inhibit ESCC progression. Moreover, patients with low expression have more postoperative relapse and metastasis than those with high expression (72.4% [97/134] vs 27.6% [37/134], *P*=0.001, Table 1), implying that TRIM24 may suppress ESCC development and metastasis. No statistical association was found between TRIM24 expression and other clinic-pathological characteristics (Table 1).

**Table 1:** Correlations between TRIM24 expression and clinicopathological characteristics in ESCC patients

Characteristics	Case (N)	TRIM24 expression		*P* value
		Low N (%)	High N (%)	
Gender				
Male	168	106 (63.1)	62 (36.9)	0.471
Female	45	31 (68.9)	14 (31.1)	
Age				
< 60	97	60 (61.9)	37 (38.1)	0.493
≥60	116	77 (66.4)	39 (33.6)	
Smoking history				
No	70	45 (64.3)	25 (35.7)	0.994
Yes	143	92 (64.3)	51 (35.7)	
Alcohol history				
No	151	94 (62.3)	57 (37.7)	0.326
Yes	62	43 (69.4)	19 (30.6)	
Tumor location				
Upper	15	8 (53.3)	7 (46.7)	0.580
Middle	142	94 (66.2)	48 (33.8)	
Lower	56	35 (62.5)	21 (37.5)	
Tumor size				
< 5	160	100 (62.5)	60 (37.5)	0.336
≥5	53	37 (69.8)	16 (30.2)	
Histological differentiation				
Well	40	26 (65.0)	14 (35.0)	0.479
Moderate	104	63 (60.6)	41 (39.4)	
Poor	69	48 (69.6)	21 (30.4)	
Tumor invasion depth				
T1-2	51	35 (68.6)	16 (31.4)	0.461
T3-4	162	102 (63.0)	60 (37.0)	
Lymph node metastasis				
N0	104	59 (56.7)	45 (43.3)	0.024
N1-3	109	78 (71.6)	31 (28.4)	
pTNM stage				
I-II	115	57 (58.3)	48 (41.7)	0.046
III	98	70 (71.4)	28 (28.6)	
Postoperative recurrence/metastasis				
No	79	40 (50.6)	39 (49.4)	0.001
Yes	134	97 (72.4)	37 (27.6)	

### Low expression of TRIM24 predicts poor prognosis in ESCC patients

**Figure 3:**
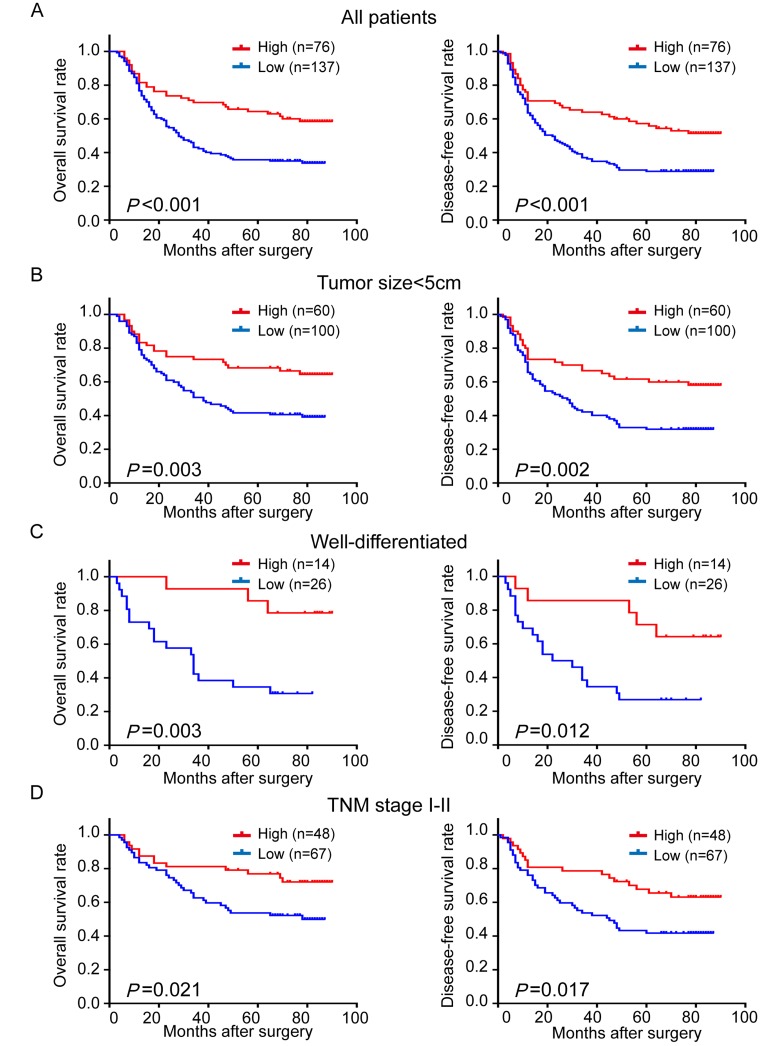
Comparison of overall survival (OS) and disease-free survival (DFS) in ESCC patients with high- and low-expression of TRIM24 protein (**A**) The survival curves (OS, left panel; DFS, right panel) of patients with high and low TRIM24 protein expressions. (**B**) – (**D**) Stratified survival analysis in patients with tumor size < 5 cm (**B**), well differentiation (**C**) and pathological TNM stage I – II (**D**).

Since TRIM24 is associated with LNM, high pTNM stage and postoperative recurrence and metastasis of ESCC patients, we reasoned that TRIM24 is correlated with survival of the patients. As expected, Kaplan–Meier survival analysis shows that patients with high TRIM24 expression have significantly better overall survival (OS) and disease-free survival (DFS) than those with low expression (*P*<0.001, *P*<0.001, respectively; Fig. 3A). Survival analysis also display that 5-year OS and 5-year DFS of patients with high TRIM24 level are 64.4% and 57.2%, compared with 35.8% and 28.9% of those with low level, respectively, which hint that TRIM24 is a tumor suppressor in ESCC patients.

To figure out whether TRIM24 expression is an independent prognostic factor in ESCC patients, univariate and multivariate analyses were conducted on TRIM24 and other clinicopathological characteristics. The univariate Cox proportional analysis reveals that the TRIM24 expression (*P* = 0.001), smoking history (*P* = 0.014), tumor size (*P* = 0.001), lymph node metastasis (*P* <0.001) and pathological TNM stage (*P*<0.001) are significant prognostic predictors for OS and DFS (Table 2). In addition, tumor invasion depth also is a significant prognostic predictor for OS (*P* = 0.012). Because tumor differentiation, location, invasive depth and lymph node metastasis are determinants of pTNM staging system (7th version), they were not included in the multivariate Cox proportional analysis. As presented in Table 2, TRIM24 expression is identified as an independent survival predictor for OS (HR, 0.519; 95%CI, 0.341-0.788; *P*=0.002) and DFS (HR, 0.584; 95%CI, 0.397-0.861; *P*=0.007), and pTNM staging system is another independent prognostic factor.

**Table 2:** Univariate and multivariate Cox proportional hazards regression models for estimating overall survival and disease-free survival

Characteristics	Univariate analysis			Multivariate analysis		
	HR	95%CI	*P* value	HR	95% CI	*P* value
**OVERALL SURVIVAL**						
Gender (female vs male)	0.699	0.440-1.110	0.129			
Age (≥60 vs <60)	1.279	0.891-1.837	0.183			
Smoking history (Yes vs No)	1.667	1.107-2.511	0.014	1.430	0.937-2.184	0.098
Alcohol history (Yes vs No)	1.272	0.870-1.860	0.214			
Tumor location (lower vs middle vs upper)	0.845	0.602-1.185	0.328			
Tumor size (≥5cm vs <5cm)	1.866	1.271-2.740	0.001	1.450	0.974-2.158	0.067
Differentiation (poor vs moderate vs well)	1.117	0.867-1.440	0.391			
Tumor invasion depth (3-4 vs 1-2)	1.835	1.145-2.940	0.012			
Lymph node metastasis (N1-3 vs N0)	3.199	2.162-4.735	<0.001			
pTNM stage (III vs II vs I)	2.426	1.747-3.371	<0.001	2.149	1.535-3.008	<0.001
TRIM24 expression (high vs low)	0.498	0.330-0.751	0.001	0.550	0.354-0.810	0.003
**DISEASE-FREE SURVIVAL**						
Gender (female vs male)	0.703	0.452-1.094	0.118			
Age (≥60 vs <60)	1.234	0.876-1.738	0.229			
Smoking history (Yes vs No)	1.568	1.070-2.299	0.021	1.423	0.959-2.110	0.080
Alcohol history (Yes vs No)	1.057	0.732-1.527	0.766			
Tumor location (lower vs middle vs upper)	0.859	0.627-1.177	0.344			
Tumor size (≥5cm vs <5cm)	1.665	1.151-2.408	0.007	1.343	0.918-1.965	0.128
Differentiation (poor vs moderate vs well)	1.137	0.894-1.446	0.294			
Tumor invasion depth (3-4 vs 1-2)	1.393	0.918-2.114	0.119			
Lymph node metastasis (N1-3 vs N0)	2.729	1.904-3.910	<0.001			
TNM stage (III vs II vs I)	2.341	1.655-3.311	<0.001	1.762	1.300-2.389	<0.001
TRIM24 expression (high vs low)	0.547	0.374-0.800	0.002	0.586	0.398-0.863	0.007

HR: hazard ratio, CI: confidence interval

Furthermore, the prognostic value of TRIM24 expression was also investigated in stratified analysis. When patients were stratified by tumor size (5 cm as a cutoff), we found that TRIM24 expression is a prognostic factor for OS (*P* = 0.003) and DFS (*P* = 0.002) in patients with tumor less than 5 cm (Fig. 3B), but not in those with tumor larger or equal to 5 cm (Supplemental Fig. 1). We also found that TRIM24 expression is a significant predictor for OS and DFS in patients with well-differentiated ESCC (OS, *P* = 0.003; DFS, *P* = 0.012; Fig. 3C) or earlier pTNM stage (I-II) (OS, *P* = 0.021; DFS, *P* = 0.017; Fig. 3D), but not in those with moderate/poor differentiation or advanced pTNM stage (III) (Supplemental Fig. 1). The above results suggest that TRIM24 mainly serves as a potential prognostic biomarker for ESSS patients in the early development stage of ESCC.

### Combined TRIM24 expression with pTNM stages markedly improves prediction of prognosis in ESCC patients

**Figure 4:**
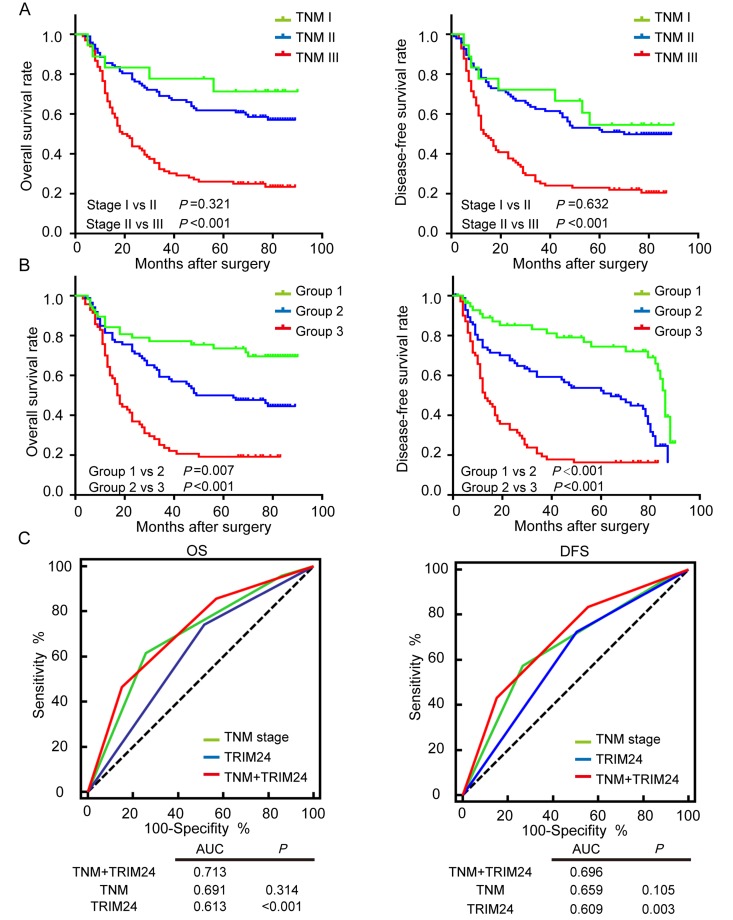
Survival prediction of ESCC patients by pTNM staging system and the combined TRIM24 protein expression with pTNM staging system (**A**) OS (left panel) and DFS (right panel) outcomes of ESCC patients were predicted by pTNM staging system. (**B**) OS and DFS outcomes of ESCC patients were predicted by the combined TRIM24 protein expression and pTNM staging system, which show that the combined risk model significantly improves survival prediction of pTNM staging system (Stage I vs II, P =0.321; Group I vs II, P =0.007). (**C**) Receiver operating characteristic (ROC) analysis compares the survival prediction of ESCC patients by pTNM staging system, TRIM24 protein expression and the combination of both. The result shows that the area under the curve (AUC) of the combined model is the largest among the three predictors, which demonstrates that predictive accuracy of the combined risk model is better than those of pTNM staging system and TRIM24 protein alone.

It is well known that TNM staging system is the main tool to predict the prognosis of malignant tumor patients. However, as shown in Figure 4A, TNM staging system inadequately predicts survival outcomes of ESCC patients with stage I and II (OS, *P* = 0.386; DFS, *P* = 0.650), which is also observed in another study [25]. Since the above result shows that TRIM24 can provide additional prognostic information, we hypothesized that TRIM24 will improve the prediction of survival in ESCC patients when it is combined with pTNM staging system. Therefore, we established a new model of combined TRIM24 and pTNM stage to calculate a new risk score for every patient. TNM stage was scored as 1 for stage I, 2 for stage II, 3 for stage III, and the expression level of TRIM24 was assigned a risk score of 0 for high expression and 1 for low expression. The final risk score for each patient is the sum of stage score and TRIM24 risk score (Range 1-4). Then all 213 ESCC patients were classified into 4 groups based on their new risk score. As expected, the survival predictive accuracy of the new risk model is much better than that of TNM staging system alone (Supplemental Fig. 2). However, the survival outcomes of Group 1 and Group 2 are not different because the sample size of Group 1 is too small (only 9 patients). In this case, we combined Group 1 and 2 into a new Group 1, and patients with combined risk score 3 and 4 were re-assigned into Group 2 and 3. As illustrated in Figure 4B, the survival outcomes of all 3 groups are significantly different. Furthermore, we employed receiver operating characteristic (ROC) analysis to compare the survival predictions of ESCC patients by pTNM staging system, TRIM24 protein expression and a combination of both. The result shows that the survival prediction of the combined risk model is the best among the three predictors. These results suggest that TRIM24 can significantly improve postoperative prognostic prediction for ESCC patients in the combined risk model.

### The relationship between TRIM24 and RARα expressions in ESCC

In this study, our findings indicate that TRIM24 functions as a tumor suppressor in ESCC, which is conflicting with some reports on other cancers [12, 26]. However, in transgenic mice models, TRIM24 is demonstrated as a tumor suppressor [16, 17, 27] and can suppress liver carcinogenesis via interacting with RARα [15].

To decipher the relationship between TRIM24 and RARα in ESCC, immunostaining was also performed on RARα in 213 ESCC samples. As shown in Figure 2A, RARα is also primarily located in the nucleus of tumor cells, and partly in the cytoplasm, which is consistent with the previous report [28]. The protein expression level in nuclear parts is significantly increased in ESCC tissues compared with the paired NCETs (*P*< 0.001, Fig. 2C). Patients were classified into high- or low-RARα expression group according to the same cutoff value (4 IRSs) as used in patients classified by TRIM24 protein. However, no significant correlation between RARα and clinicopathological characteristics was found except for gender (*P* = 0.004, Supplemental Table 2). Moreover, RARα expression also is not associated with OS (*P* = 0.223, Supplemental Fig. 3A) and DFS (*P* = 0.770, Supplemental Fig. 3A). Surprisingly, the expression level of RARα protein has no association with TRIM24 expression level according to Pearson's correlation analysis (data not shown), which suggests that RARα protein may not be correlated with TRIM24 protein in ESCC.

## DISCUSSION

The tripartite motif superfamily, including TRIM24, has been established to be involved in a broad range of biological process including cell differentiation, development and homoeostasis, as well as several pathological conditions, such as Mendelian genetic diseases, cancer development and viral infection [7]. TRIM24, initially identified as a fusion partner of the B-raf protein in the oncoprotein T18 found in mouse hepatocellular carcinoma [29], has been reported to be overexpressed in several kinds of cancers as afore-mentioned, which indicates that TRIM24 plays an oncogenic role in carcinogenesis. In contrast to these reports, however, our study demonstrates for the first time that TRIM24 is reduced in ESCCs when compared with NCETs, as judged by qRT-PCR, Western blot and immunohistochemistry, which suggests that TRIM24 may be a tumor suppressor in ESCC.

Proteins located in different subcellular compartments must play a different function. In the current study, immunohistochemical staining shows that TRIM24 protein is predominantly localized in the nuclei of ESCC cells and NECT cells, suggesting that it may be involved in the transcriptional process, which is in accordance with other reports [12, 30]. However, TRIM24 protein is located not only in the nucleus but also in the cytoplasm of prostate cancer cells, as displayed by immunofluorescence [31], indicating that TRIM24 protein plays different roles in different cancers. More important, our study shows that lower expression level of TRIM24 protein is associated with more lymph node metastasis, advanced pTNM stage and postoperative recurrence/metastasis, signifying that TRIM24 protein mainly functions as tumor suppressor in early development of ESCC and loss of TRIM24 protein may result in the progression of ESCC. Furthermore, survival analysis reveals that patients with decreased level of TRIM24 have a poor survival rate and that TRIM24 is an independent prognostic factor in ESCC. In stratified analysis, TRIM24 protein is a significant prognostic factor in patients with tumor size <5 cm, early clinical stage, and well differentiation, but not in patients with tumor size ≥5 cm, advanced clinical stage, and moderate or poor differentiation, indicating that TRIM24 protein as a prognostic factor is more significant in patients with early stage than in those with advanced stage. All of the evidence demonstrates that TRIM24 functions as a tumor suppressor in ESCC.

Studies show that TRIM24 protein plays different roles in different cancers, suggesting that TRIM24 protein has complex functions in development and progression of cancer. An earlier study defined TRIM24 as an E3-ubiquitin ligase that could degrade p53 protein, and loss of TRIM24/*bonus* would result in increased p53 expression and p53-dependent apoptosis in Drosophila and p53-postive MCF7 human breast cancer cells [8]. However, reduced TRIM24 expression causing p53 upregulation has not been confirmed in human breast cancer tissues and head and neck squamous cell carcinoma samples [12, 30]. Moreover, a recent report reveals that TRIM24 and other genes can bind mutant p53 and shift its conformation to a wild type form, which is a novel mechanism of maintenance of genomic integrity by p53 [32]. Importantly, in a mouse model with cre-mediated excision of exon 4 of TRIM24 (TRIM24*^dlE4/dlE4^*), Khetchoumian K et al found that deficiency of TRIM24 may lead to continuous hepatocytes proliferation, spontaneous hepatocarcino-genesis, and increased susceptibility to chemically induced HCC [15, 16, 27]. Moreover, in another similar mouse model with liver-specific complete deletion of TRIM24 (TRIM24*^hep/hep^*), hepatic lipid-filled lesions, steatosis, hepatic injury, fibrosis and hepatocellular carcinoma without high-fat diet are observed [17]. These studies indicate that TRIM24 functions as a tumor suppressor in hepatocarcinogenesis in mouse. Mechanically, TRIM24 inhibits RARα dependent transcription to negatively regulate IFN/ STAT signaling pathway in the hepatocarcinogenesis of mice [15, 33].

RARα is one of nuclear retinoic receptors that mediate a spectrum of pleiotropic functions of retinoids [34]. Previous studies demonstrated that the expression level of RARα is increased in various premalignant and malignant lesions of esophagi [23, 24, 35]. Consistent with those previous reports, our results show that the expression level of RARα protein is significantly upregulated in ESCC tissues compared with distant normal esophageal tissues, suggesting it may be involved in esophageal tumorigenesis. Interestingly, female patients with ESCC have significantly lower expression level of RARα protein compared with male patients (Supplemental Table 2), implying that RARα mainly takes part in ESCC carcinogenesis of male patients. No statistically significant correlation is observed between RARα expression level and the prognosis of ESCC patients, which is in accordance with another report on ESCC patients [36].

In addition, Pearson's correlation analysis indicates that there is no significant relationship between TRIM24 and RARα protein expression levels. A similar result was observed in non-small cell lung cancer with immunohistochemistry [26], and down-regulation of TRIM24 in head and neck squamous cell carcinoma (HNSCC) cells (WSU-HN6 cells) did not result in high mRNA expression level of RARα [30]. These results indicate that TRIM24 is not correlated with RARα in the development and progression in these cancers, which is different from the TRIM24 that suppresses RARα-dependent transcription during hepatocarcino-genesis in mice. Taking our results together with those of previous studies, we supposed that TRIM24 may function as a tumor suppressor in ESCC independent of RARα-dependent transcription.

In conclusion, our study reveals that TRIM24 mRNA and protein are reduced in ESCC tissues and the reduced TRIM24 protein is associated with LNM, advanced TNM stage and post-operative recurrence and metastasis; more important, TRIM24 protein level has been proven to be an independent prognostic factor for OS in ESCC patients. These results indicate that TRIM24 functions as a tumor suppressor and is a potential biomarker for prognosis in ESCC patients.

## MATERIALS AND METHODS

### Patients and tissues

The formalin-fixed paraffin-embedded (FFPE) ESCC tissues were obtained from 213 patients who underwent esophagectomy between January 2008 and December 2009 in Sun Yat-Sen University Cancer Center. All patients were included according to as the following criteria: i) Not receiving any chemotherapy and/or radiotherapy before surgery; ii) R0 resection, which means that no tumor tissue at the surgical margins of the resected specimens was found by postoperative pathology examination; iii) Esophageal squamous cell carcinoma diagnosed by pathology; iv) No distant metastases before surgery, including supraclavicular and paraaortic lymph node metastases. The cohort patients consisted of 168 males (78.9%) and 45 females (21.1%), aging from 34 to 88 years (mean age: 60 years old). The follow-up time ranged from 3 to 90 months with a median of 45 months. Overall survival (OS) was defined as from the date of surgery to the date of death or last follow-up, and disease-free survival (DFS) time was from the date of surgery to the date of recurrence, metastasis, or last follow-up. Clinical and pathological tumor staging was performed according to the 7^th^ edition of the TNM classification of the International Union against Cancer (UICC).

Another forty-two fresh paired ESCC tissues and adjacent noncancerous esophageal tissues (NCET) that were at least 2cm far from the tumor tissues were collected from the patients who had undergone esophagectomy at the Cancer Center from June 2012 to December 2013. Written informed consent was obtained from all the patients before the surgery, in which patients agreed to use their medical record data and tissue samples for the research. This study was reviewed and approved by the medical ethics committee of Sun Yat-Sen University Cancer Center.

### Isolation of total RNA and real-time reverse transcription quantitative PCR (RT-qPCR)

The total RNA from tumor tissues and the paired non-tumor samples was extracted by TRIzol reagents following the manufacturer's instructions (Invitrogen life Technologies, Carlsbad, CA, USA). RNA was evaluated in a NanoDrop^TM^ 2000 spectrophotometer (ThermoFisher Scientific, Waltham, MA, USA). All RT-qPCR reactions were performed as described previously [37]. Briefly, 1 μg of total RNA was reversely transcribed into cDNA using the Moloney Leukemia Virus Reverse Transcriptase (A5001; Promega, Madison, WI, USA). Quantitative real-time PCR was performed on Roche PCR machine in a total 15 μL volume of reaction mixture consisting of 7.5 μL of GoTaq® qPCR Master Mix (A6001; Promega), 0.25 μL (10μmol/L) of each primer, 1 μL cDNA template, and 6μL nuclease-free water. PCR protocol is as follows: 95°C for 120s, 45 cycles of 95°C for 15s, 60°C for 60s, followed by a dissociation stage. The detection for each sample was repeated in triplicate. To calculate the relative level of TRIM24 mRNA expression in tumor and paired adjacent normal tissues, the comparative CT method (2^−**ΔΔCT**^) was performed and GAPDH was detected as the reference gene. The primers used in the RT-PCR were as follows: TRIM24 forward, 5^’^-GGAGTCATTCGTTGCCCAGT-3^’^; reverse, 5^’^-TTCTGCGTTGTCCTCACAGC-3^’^; amplification fragment length: 147bp. GAPDH forward, 5^’^-CTCCTCCTGTTCGACAGTCAGC-3^’^; GAPDH reverse, 5^’^-CCCAATACGACCAAATCCGTT-3^’^; amplification fragment length: 205bp.

### Protein extraction and Western blot analysis

19 ESCC and matched NCET tissues were selected randomly from all the fresh samples, and homogenized to extract protein in a RIPA lysis buffer containing 1 mM phenylmethylsulphonyl fluoride (PMSF). Protein concentration was measured by BCA protein assay. After mixing with 4×SDS sample buffer, 50μg of the protein samples were separated in 8% SDS-PAGE and then transferred onto polyvinylidene difluoride (PVDF) membranes. Then membranes were blocked with TBST containing 5% skimmed milk and incubated sequentially with the primary antibodies against TRIM24 (1:1000 dilution) (14208-1-AP; Proteintech, Chicago, IL, USA) and GAPDH (1:1000 dilution) (#5174; Cell Signaling Technology, USA) at 4°C overnight. After three 10-minute washes with TBST, the membranes were incubated with HRP-conjugated secondary antibodies (dilution for 1:5000) (Jackson Immunoresearch Inc, PA, USA) for about 1 hour at room temperature. The immunocomplexes were detected by an enhanced chemiluminescence system (ECL; cell signaling), and the gray value of the protein bands was measured by using Image J software (http://rsb.info.nih.gov/).

### Immunohistochemistry

Surgically resected esophageal tumor specimens were fixed with 10% formalin, embedded in paraffin, and sectioned into 4mm-thick slide. The section was immersed twice in xylene for 10 minutes each. After being rehydrated in a series of graded alcohol (100%, 95%, 85%, 75%, 50%) for 5 minutes each, the section was washed with Phosphate Buffered Saline (PBS) three times for 5 min each. Endogenous peroxidase activity was blocked by 3% H_2_O_2_ solution for 10 minutes, and then the sections were rinsed again with PBS. The sections were then put in 0.01M citrate buffer and heated for 20 min in a microwave to retrieve antigen. When the sections were cool to room temperature, they were blocked with 10% fetal bovine serum (FBS) for 30 min to prevent nonspecific staining. After being washed with PBS, tissue sections were incubated with TRIM24 rabbit polyclonal antibody (1:1000 dilution) (14208-1-AP; Proteintech, Chicago, IL, USA) or RARα rabbit polyclonal antibody (1:150 dilution) (sc-551; Santa Cruz Biotechnology, Santa Cruz, CA, USA) overnight at 4°C. Negative control was established by omitting the primary antibody. After washing, the sections were incubated with HRP polymer conjugated Pika universal antibody (Dako, Glopstrup, Denmark) for about 30minutes at room temperature, followed by diaminobenzidine (DAB) to develop color reaction. Hematoxylin was used for counterstaining, and then the sections were dehydrated in ethanol before mounting.

Two experienced pathologists who were blinded to the clinical information evaluated the immunohisto-chemistry staining independently. The staining intensity (SI) of TRIM24 protein was defined as 0 (negative), 1 (weak), 2 (moderate), 3 (strong), and the percentage of positive staining (PP) was assigned as 1 (1-25%), 2 (26-50%), 3 (51-75%), 4 (76-100%). The final immunoreactive score (IRS) was calculated by the multiplication of SI and PP, from 0 to 12. The patients were stratified into a low expression group (≤ 4) or a high expression group (> 4) based on the final scores [26].

### Statistical analysis

The SPSS 16.0 software package (Chicago, IL, USA) and Graph Pad Prism 6 software (www.graphpad.com) were used for all the statistical analysis in this study, and *P* value <0.05 was considered statistically significant. The Chi-square test was performed to analyze the correlation between TRIM24 or RARα expression level and the various clinicopathological parameters. Comparison of mRNA and protein expression levels of TRIM24 and protein level of RARα in ESCCs and the paired NCETs was carried out using paired-sample *t* test. Pearson's correlation analysis was used to examine whether there is a relationship between the expression level of TRIM24 and RARα. In order to estimate the overall survival (OS) and disease-free survival (DFS), the Kaplan-Meier curves and the log-rank test were used. Univariate and multivariate Cox proportional hazards regression analyses were performed to evaluate the potential prognostic biomarkers and clinical factors.

## SUPPLEMENTAL MATERIAL TABLES AND FIGURES


