# Supplementary material for: Clinical significance and prognostic value of TRIM24 expression in esophageal squamous cell carcinoma
Source: Aging (Albany NY). 2016 Sep 28;8(9):2204–17. doi: 10.18632/aging.101037 (PMC5076458; doi:10.18632/aging.101037)
Supplement: Supplementary file 1 [file aging-08-2204-s001.pdf]

SUPPLEMENTAL MATERIAL

Supplemental Table 1. Correlations between TRIM24 mRNA expression and clinico-pathological characteristics in ESCC patients

	Case (N)	TRIM24 expression		*P* value
		Low N (%)	High N (%)	
Gender				
Male	36	17 (47.2)	19 (52.8)	0.659[a]
Female	6	4 (66.7)	2 (33.3)	
Age				
< 60	20	11 (55.0)	9 (45.0)	0.537
≥60	22	10 (45.5)	12 (54.5)	
Smoking history				
No	14	8 (57.1)	6 (42.9)	0.513
Yes	28	13 (46.4)	15 (53.6)	
Alcohol history				
No	22	13 (59.1)	9 (40.9)	0.217
Yes	20	8 (40.0)	12 (60.0)	
Tumor location				
Upper	3	3 (100.0)	0 (0.0)	0.367[b]
Middle	26	12 (46.2)	14 (48.3)	
Lower	13	6 (46.2)	7 (53.8)	
Tumor size				
< 5	21	9 (42.9)	12 (57.1)	0.355
≥5	21	12 (57.1)	9 (42.9)	
Histological differentiation				
Well	5	4 (80.0)	1 (20.0)	0.101[b]
Moderate	25	14 (56.0)	11 (44.0)	
Poor	12	3 (25.0)	9 (75.0)	
Tumor invasion depth				
T1-2	7	4 (57.1)	3 (42.9)	1.000
T3-4	35	17 (48.6)	18 (51.4)	
Lymph node metastasis				
N0	21	8 (38.1)	13 (61.9)	0.123
N1-3	21	13 (61.9)	8 (38.1)	
pTNM stage				
I-II	21	8 (38.1)	13 (61.9)	0.123
III	21	13 (61.9)	8 (38.1)	

[a]correction for continuity; [b] Fisher exact probability

Supplemental Table 2. Correlation between the expression of RARα and clinicopathological characteristics in ESCC patients

Characteristics	Case No (N)	RARα expression		*P* value
		Low N (%)	High N (%)	
Gender				
Male	168	87 (51.8)	81 (48.2)	0.004
Female	45	34 (75.6)	11 (24.4)	
Age				
< 60	97	49 (50.5)	48 (49.5)	0.090
≥60	116	72 (62.1)	44 (37.9)	
Smoking history				
No	70	41 (58.6)	29 (41.4)	0.716
Yes	143	80 (55.9)	63 (44.1)	
Alcohol history				
No	151	87 (57.6)	64 (42.4)	0.710
Yes	62	34 (54.8)	28 (45.2)	
Tumor location				
Upper	15	8 (53.3)	7 (46.7)	0.151
Middle	142	75 (52.8)	67 (47.2)	
Lower	56	38 (67.9)	18 (32.1)	
Tumor size				
< 5	160	88 (55.0)	72 (45.0)	0.355
≥5	53	33 (62.3)	20 (37.7)	
Histological differentiation				
Well	40	25 (62.5)	15 (37.5)	0.719
Moderate	104	58 (55.8)	46 (44.2)	
Poor	69	38 (55.1)	31 (44.9)	
Tumor invasion depth				
T1-2	51	29 (56.9)	22 (43.1)	0.993
T3-4	162	92 (56.8)	70 (43.2)	
Lymph node metastasis				
N0	104	60 (57.7)	44 (42.3)	0.799
N1-3	109	61 (56.0)	48 (44.0)	
pTNM stage				
I- II	115	68 (58.1)	47 (40.9)	0.458
III	98	53 (54.1)	45 (45.9)	
Recurrence/ metastasis				
No	79	47 (59.5)	32 (40.5)	0.543
Yes	134	74 (55.2)	60 (44.8)	

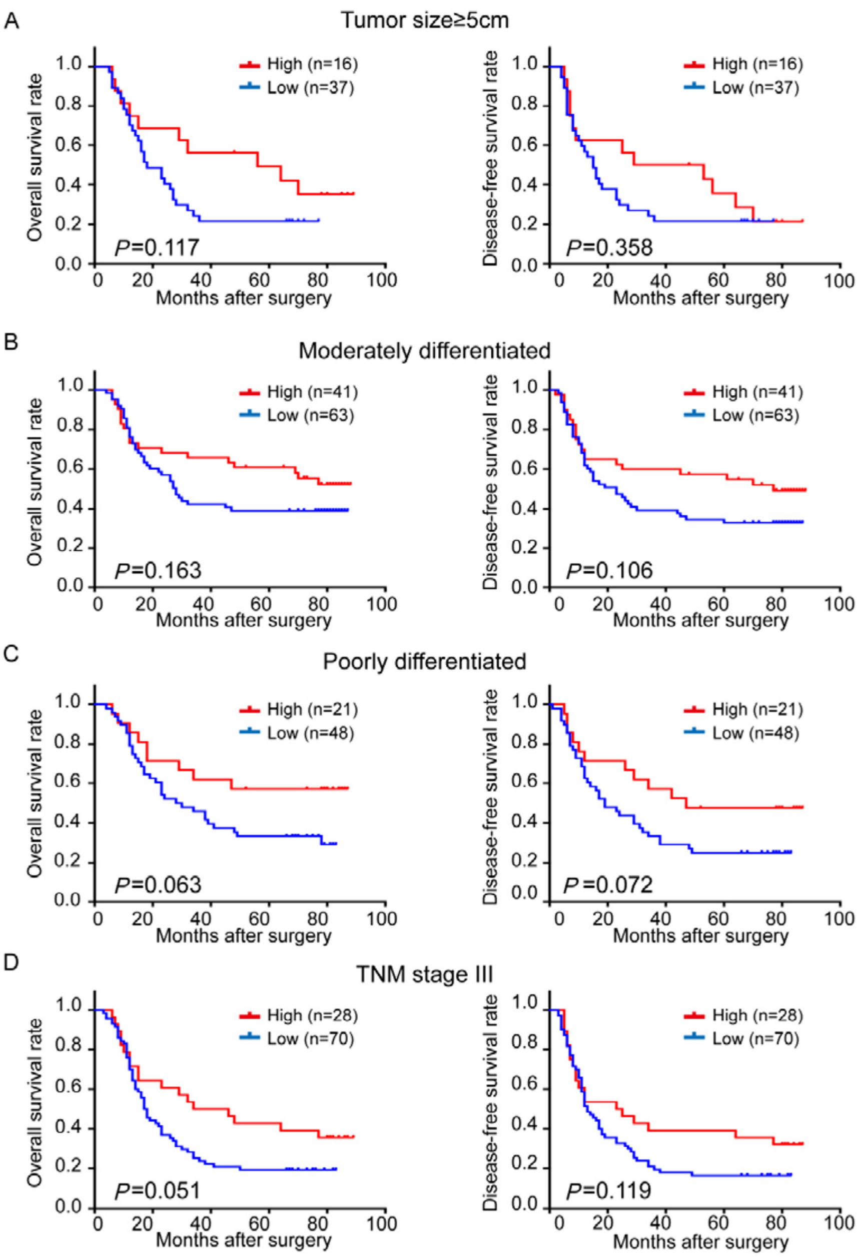

Supplemental Figure 1. Overall survival and disease-free survival in the stratified ESCC patients with high- and low-expression of TRIM24 protein. All the 213 patients were stratified by tumor size, tumor differentiation and pTNM stage. However, no statistical association was found between the expression level of TRIM24 and the prognosis in the subgroups of patients with tumor size ≥5 cm (**A**), moderate differentiation (**B**) and poor differentiation (**C**) and pTNM stage III (**D**).

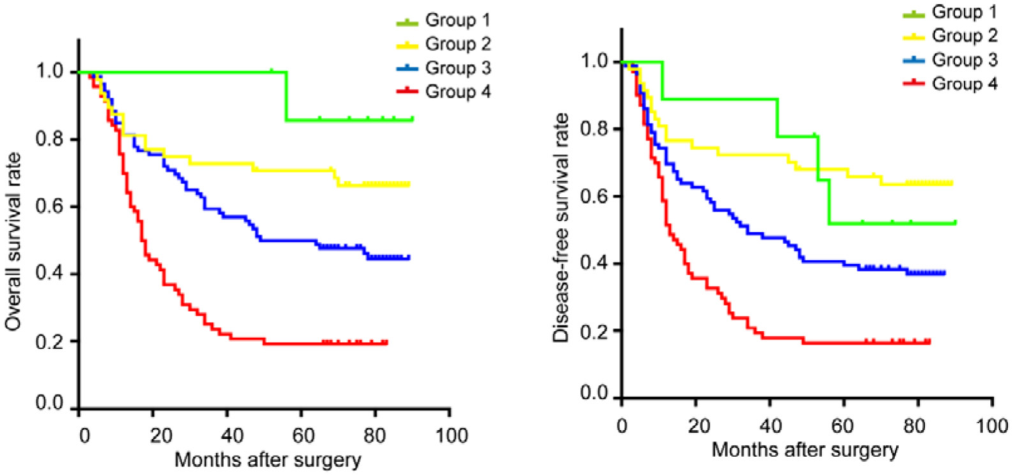

Group	*P*
1 vs 2	0.210
1 vs 3	0.028
2 vs 3	0.035
3 vs 4	<0.001

Group	*P*
1 vs 2	0.772
1 vs 3	0.208
2 vs 3	0.006
3 vs 4	<0.001

Supplemental Figure 2. Overall survival and disease-free survival curve that predicted by the combination of TRIM24 protein and pTNM staging system. The combined risk model that classifies all the ESCC patients into four groups is more accuracy in predicting survival than pTNM staging system alone.

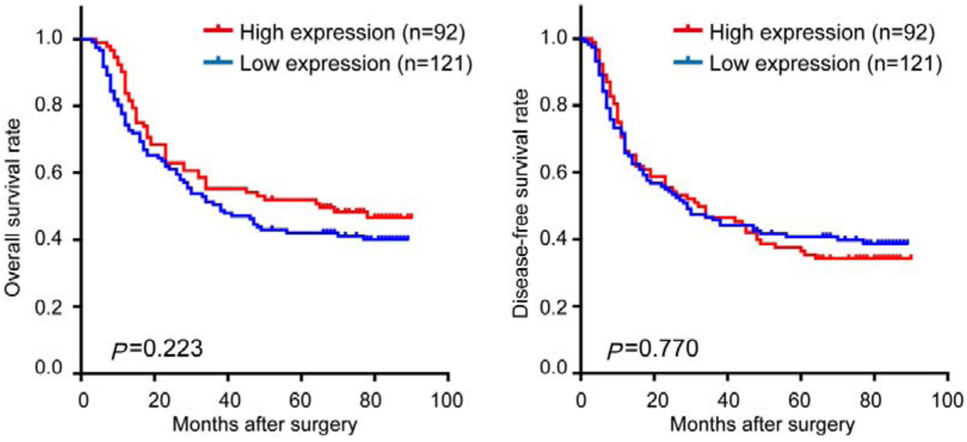

Supplemental Figure 3. Kaplan–Meier survival curve of overall survival and disease-free survival in ESCC patients with high- and low-expression of RARα protein. The expression of RARα protein level was not correlated with the overall survival rate (log-rank, P = 0.223) and disease-free survival rate (log-rank, P = 0.770).
